# Experiments

**Published:** 1800-09

**Authors:** Charles Darwin


### *Experiments, by Dr.* Charles Darwin.

[Continued from p. 108, of our laft.]

Exp. XVII. To a fmall quantity of *coagulable lymph*, obtained by wafhing away the red globules from fome craffamentum of blood, I added about fifty drops of *acid* of *vitriol*; which changed the colour of the lymph to a reddifh brown, but did not apparently diffolve any of it.

Exp. XVIII. To No. xvii. about an ounce of water was added without producing any obfervable change in the fluid, but the lymph after this feemed more denfe than before Experiment xvii.

Exp. XIX. One drachm of *cauftic lixivium* took five hours to diffolve the greater part of four grains of *coagulable lymph*; the remainder of the lymph had acquired a brown red colour, and was fofter than before it was added to the alkali.

Exp. XX. Water added to xix. produced a feparation of a whitifh fediment.

Exp. XXI. Equal parts of *ferum* and *cauftic lixivium* eafily united, and fuffered no change on the addition of eight or ten times their quantity of water; though after ftanding fome hours the mixture depofited a flight fediment.

Exp. XXII. Twenty-five drops of *vitriolic acid* added to one drachm of *ferum* converted it into a whitifh curdled matter.

Exp. XXIII. Water being added to No. xxii. the whitifh curdled matter was eafily difperfed through it; but foon formed a cloud near the top of the glafs. On the addition of more ferum to this mixture, it was converted into a fimilar matter.

Exp. XXIV. One drachm of *nitrous acid*, after fome time, diffolved three grains of *coagulable lymph*, with fumes and froth.

Exp. XXV. Water added to this, produced a dilute *green fluid*.

Exp. XXVI. *Acid of nitre* diffolved *mucus*, and with water occafioned a turbid dirty-coloured fluid.

Exp. XXVII. A faturated folution of *corrofive fublimate* coagulated *mucus* into a harder white material.

Exp. XXVIII. (This and the following Experiments were tried on Pus from a fuppurated mamma.) *Pus* diffufed readily through *pure water*, forming a white milky opake fluid, but after fome length of time fell down to the bottom of the glafs, where it had no great vifcidity; the fluid above it was for fome hours of a reddifh yellow colour, but became at laft colourlefs. By agitation the pus was again diffufible, but did not remain fo long fufpended as the firft time; and the fluid was immediately after its precipitation clear and colourlefs.

Exp. XXIX. *Acid of vitriol in fmall quantity* exhibited the fame appearances with this *pus* from a fuppurated mamma, as in No. 1, &c.

[ To be continued. ]

